# A new long-term sampling approach to viruses on surfaces

**DOI:** 10.1038/s41598-021-96873-9

**Published:** 2021-09-02

**Authors:** Julia Sommer, Martin Bobal, Birgit Bromberger, Patrick-Julian Mester, Peter Rossmanith

**Affiliations:** 1 Unit of Food Microbiology, Institute of Food Safety, Food Technology and Veterinary Public Health Department for Farm Animals and Public Health in Veterinary Medicine, University of Veterinary Medicine, Veterinärplatz 1, 1210 Vienna, Austria; 2 Joint BioEnergy Institute, Lawrence Berkeley National Laboratory, Berkeley, USA; 3 Present Address: Vetfarm and Clinical Unit of Herd Health Management for Ruminants, Department for Farm Animals and Public Health in Veterinary Medicine, University of Veterinary Medicine, Kremesberg 14, 2563 Pottenstein, Austria

**Keywords:** Microbiology, Molecular biology

## Abstract

The importance of virus disease outbreaks and its prevention is of growing public concern but our understanding of virus transmission routes is limited by adequate sampling strategies. While conventional swabbing methods provide merely a microbial snapshot, an ideal sampling strategy would allow reliable collection of viral genomic data over longer time periods. This study has evaluated a new, paper-based sticker approach for collection of reliable viral genomic data over longer time periods up to 14 days and after implementation of different hygiene measures. In contrast to swabbing methods, which sample viral load present on a surface at a given time, the paper-based stickers are attached to the surface area of interest and collect viruses that would have otherwise been transferred onto that surface. The major advantage of one-side adhesive stickers is that they are permanently attachable to a variety of surfaces. Initial results demonstrate that stickers permit stable recovery characteristics, even at low virus titers. Stickers also allow reliable virus detection after implementation of routine hygiene measures and over longer periods up to 14 days. Overall, results for this new sticker approach for virus genomic data collection are encouraging, but further studies are required to confirm anticipated benefits over a range of virus types.

## Introduction

The importance of virus disease outbreaks is of growing public concern. While viral epidemics and pandemics caused by Ebola, MERS-CoV and the current SARS-CoV-2 are most likely temporary events^1,2^, food-borne disease outbreaks as well as annual influenza outbreaks pose enduring threats to human health^3,4^. However, regardless of the viral pathogen, virus detection and determination of transmission routes are still poorly understood, largely because of inadequate sampling and monitoring tools. Until now the gold standard for virus detection, especially for infectious viruses, has been the plaque assay^5,6^. However, for verification purposes, growth-dependent assays are cost and time intensive and are not suitable for all viruses^7^. Consequently, there has been a drive for other, growth-independent detection methods for rapid virus detection^7,8^.

In general, virus detection in the food industry and healthcare settings is mostly performed by molecular methods, especially polymerase chain reaction (PCR) techniques^9,10^. However, swab sampling is still accepted as the method of choice^11^. Extensive surface sampling is not only relevant for detecting viral pathogens, but also for the demonstration of contamination routes, contamination sources and for monitoring persistent viruses in different environments^12–14^.

As viruses are able to endure in bacterial biofilms and remain infectious over long periods, their persistence in food facilities, hospitals and other institutions has been suspected to be a recurring cause of disease outbreaks^15,16^. Although swabbing methods are easy to perform, much effort has been expended to obtain more than merely a momentary, microbial snapshot from surfaces. Primarily, the sampling time point is not trivial as flushing, cleaning and disinfection can blur the actual viral load in multiple ways^17^. Since hygiene measures are essential to minimize pathogen transmission, ideally sampling approaches must overcome this limitation, which is essentially information loss^17^. Moreover, long-term virus monitoring strategies have likewise been neglected. Resolution of these major limitations requires novel, innovative sampling approaches to enable virus traceability and, simultaneously, increased safety.

**Figure 1:**
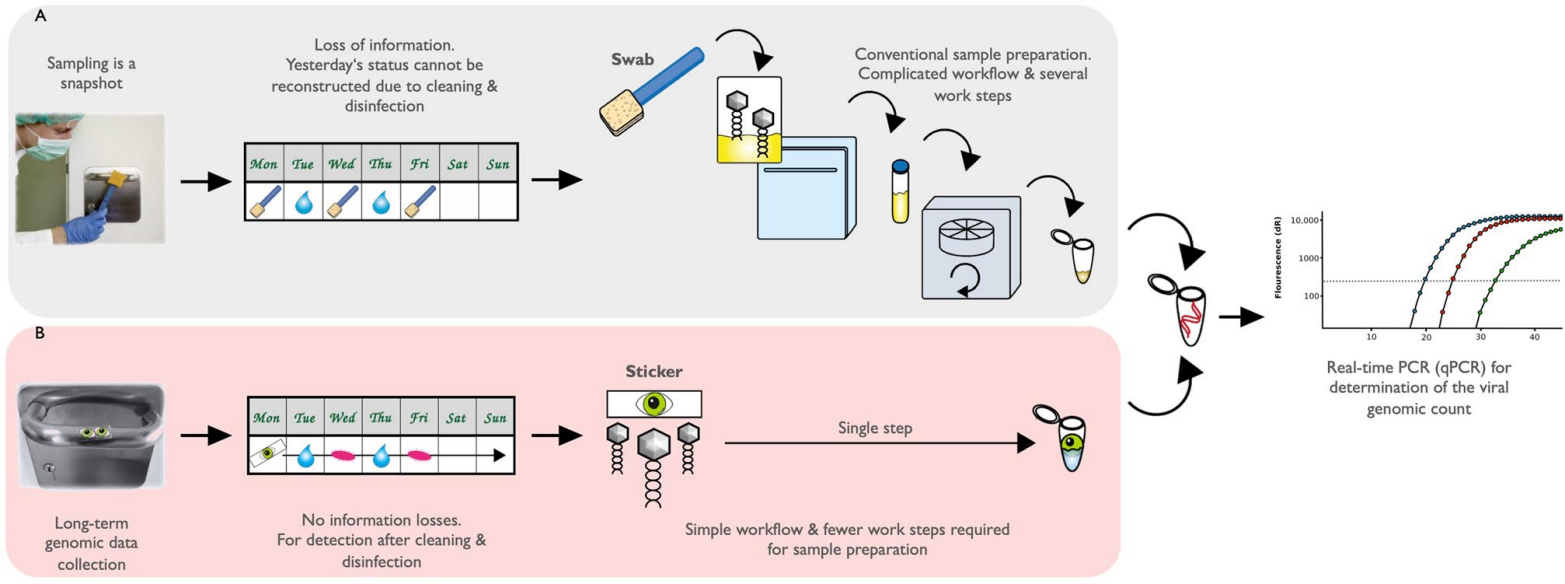
This figure depicts surface sampling procedures using common swabs (**A**) and paper-based adhesive stickers (**B**). While conventional swabbing before and after each hygiene step is required for reliable results long-term, the sticker approach requires only one self-adhesive sticker, which can be easily applied to a wide variety of surfaces. The sticker approach (**B**) also provides a simple workflow and fewer steps than conventional swab sampling (**A**) prior to qPCR analysis.

This study focused on the evaluation of a new paper-based sticker approach for detection of viruses on surfaces over extended time periods. Recently Bobal and Witte et al.^18^ demonstrated the advantages of this sticker technique for detection and monitoring of bacterial pathogens on surfaces, including door handles. However, its utility for virus surface sampling has yet to be investigated. The main advantage of this new technique is its simplicity, as shown in Fig. 1. The novel, essential feature of one-side adhesive paper-based stickers is that they can be easily applied to the majority of surfaces, where they collect viral particles that would be otherwise have been transferred onto that surface. Permanent application of the sticker to surfaces permits collection of viral genomic data over extended time periods, during which routine hygiene measures can continue to be practiced. Unlike common swabs, this new sampling approach does not reflect the current microbial load on a surface at a certain point of time, but due to its collection capacity it is suitable for long-term monitoring issues. Additionally, the sticker microbial sample can be easily extracted in one step (shown in Fig. 1), compared to conventional swab sampling that necessitates several preparation steps prior to detection using qPCR. Therefore, this new sampling approach offers a new and novel way of virus sampling and monitoring with minimized effort. However, in order to exploit the simplicity and expeditious characteristics of this sampling, a detailed analysis of sticker properties for virus collection is required. We evaluated recovery of infectious virus particles, the linear recovery properties of stickers, the recovery rate of viral genomic material after implementation of different hygiene measures such as cleaning and disinfection, and stability for up to 14 days.

## Results and discussion

Until now virus transmission routes have been difficult to track due to a lack of adequate sampling strategies. Most of these employ swabs for virus uptake, which merely capture a momentary snapshot, and which are strongly influenced by hygiene measures that contribute to lost microbial information. Although a momentary snapshot of a surface is sufficient for virus detection, this type of sampling does not allow an all-encompassing recording of data over a longer period of time. So far novel long-term surface monitoring strategies and sampling materials for virus data collection are being rarely described^19^, this study focuses on evaluation of a new sampling approach that is both simple and based on existing material—the plain paper-based sticker. Both microbiological and molecular biological methods have been used to evaluate the benefits of this new paper-based sampling material for virus collection. As to this date no comparable sampling and monitoring approach to the recently invented paper-based sticker exists, we had to compare the routine cotton swabs and the sticker approach to each other, although a direct comparison is unsuitable, due to the fact that the new sticker approach will collect viral particles that would be otherwise have been transferred onto that entire surface of interest. In order to ensure an adequate evaluation of the new paper-based sticker, a comparison of a well-known sampling material was necessary. As the cotton swab is one of the best-known sample materials, it was chosen to clearly define the benefits of the new sticker sampling method and to determine its limitations. However, a clear separation of both methods is important, as their application and relating thereto-informative value strongly related to the question and issue posed by the user.

### Recovery of infectious virus particles and determination of the linear and long-term monitoring properties of stickers

**Figure 2:**
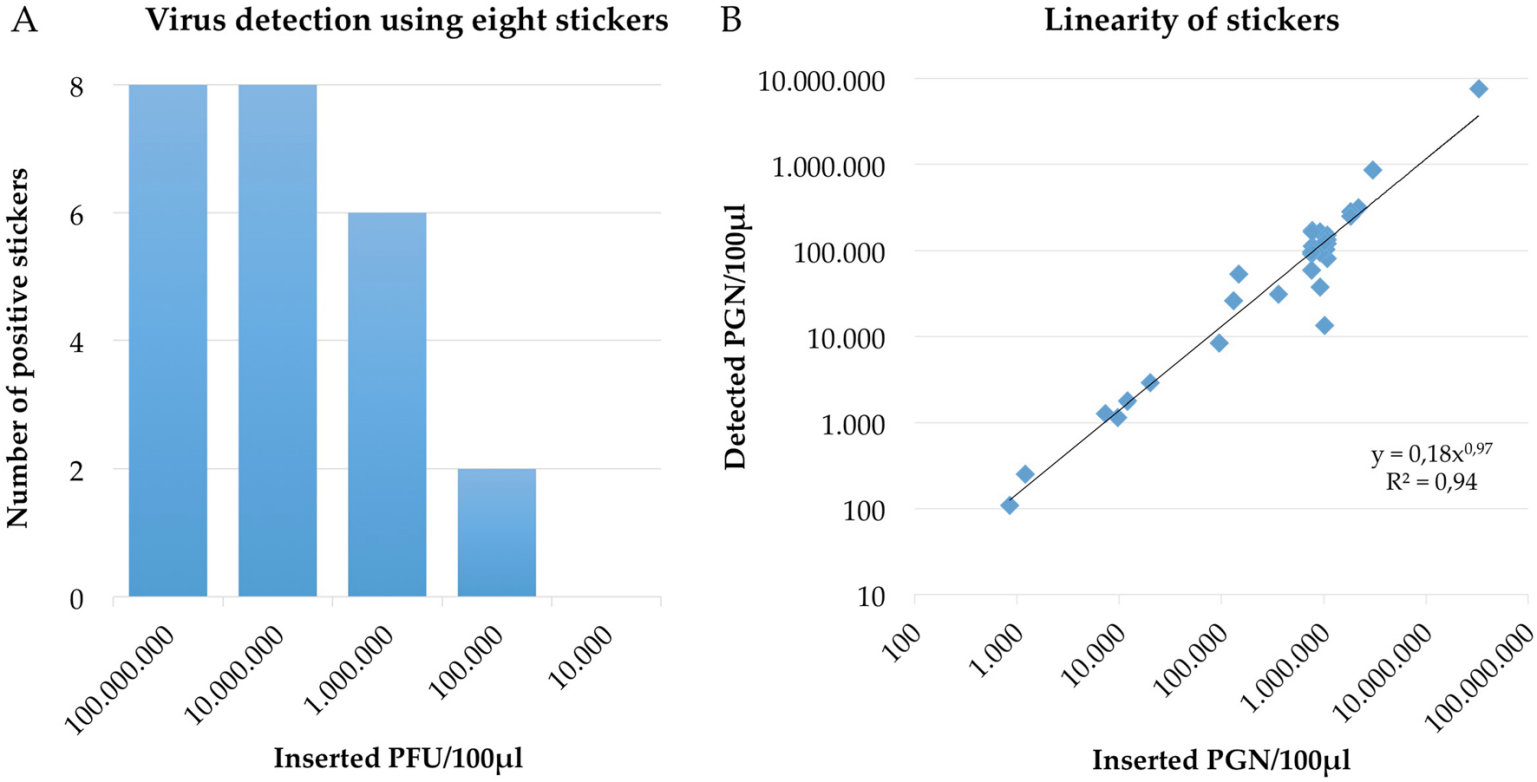
(**A**) The number of positive findings of eight sticker values and swab values determined by plaque assay from four independent experiments performed in duplicate. Virus titer concentrations ranging from 10^8^ to 10^4^ were used for artificial, direct contamination of paper-based stickers and routine cotton swabs. (**B**) The quantification of artificially contaminated plain-paper stickers (n = 32) with virus P100. The sampling material was contaminated with known virus titers ranging from 10^8^ to 10^2^ PGN/100 µL to cover a broad recovery range. The x-axis plots the inserted quantity of viral genomic matter (PGN/100 µL) and the y-axis plots the recovered viral genomic matter from respective sampling materials.

We initially compared the paper sticker sampling methods for infectious virus particles using plaque assays, the contemporary diagnostic gold standard^5,6^. The quantity of infectious virus particles (plaque forming units (PFU)/100 µL) of artificially contaminated stickers and swabs was determined. Results presented in Table S1 indicate that neither cotton swabs nor stickers achieved reliable recovery rates of infectious viral particles over a broad concentration range. Indeed, the 8/8 positive stickers or swabs score was only achievable using the two highest concentrations of virus solutions (10^8^ and 10^7^ PFU/100 µL); results presented in Fig. 2A and Table S1. This outcome is not surprising as the difficulties experienced in determining infectious virus particles from swab material has already been described in the literature, whereas the quantity of recovered infectious particles correlates with sampling material characteristics, such as electrostatic charge and others^20,21^. As the binding strength of a virus depends on its isoelectric point, the interaction between virus and sampling material must be determined for each virus individually^20,21^. Alternatively, poor recovery can be a consequence of the inactivating effect of the sampling material itself; inactivation of virus particles on filter papers has already been demonstrated by Picard-Meyer and Cliquet^22^. Nevertheless, the interactions of virus P100 and sticker material was beyond the scope of this study.

Although sampling materials recovered similar quantities of infectious virus particles, growth-dependent verification is disadvantaged in respect of its detection limit. The detection limit for virus P100 with the plaque assay was approximately 10^4^ PFU/100 µL (Fig. 2; Table S1). This is a relatively high concentration and it prompted us to use qPCR instead.

To investigate the broad range linearity of detection of this new approach, the stickers were spike-inoculated with virus concentrations ranging from 10^8^ to 10^2^ phage genome number (PGN)/100 µL and the viral load was determined using qPCR. The same protocol was used for the swabs, which were directly compared. As shown in Fig. 2B the stickers achieved a coefficient of determination of 0.94, which is in good accordance with 0.98 obtained for swabs (Figure S1). This represents good linear detection properties over a broad viral concentration range for both sampling materials. Stickers and swabs also achieved similar virus recovery rates over a broad virus concentration range; stickers had a mean recovery of 15% and swabs 18% (Table S2), also at lower concentrations of about 10^2^ PGN/100 µL^23^. These outcomes are highly relevant as they demonstrate that stickers have comparable sensitivity to the current virus sampling system.

As Cardona-Ospin et al.^24^ have demonstrated the efficacy of paper-based filters for preservation of viral RNA, we investigated the long-term characteristics of stickers and whether they were suitable for collection of viral genomic matter over longer time periods. Stickers were inoculated with a known virus suspension and the detectable genomic viral load after 0, 1, 3, 7 and 14 days was determined. Results are presented in the supplement Figure S2 and indicate that the viral load remains constant until day 7. After 14 days of incubation a slight decrease could be observed. However, these results confirm that long-term monitoring of viral genomic loads is possible with paper-based stickers.

### Viral DNA recovery from stickers and the effect of hygiene measures

**Figure 3:**
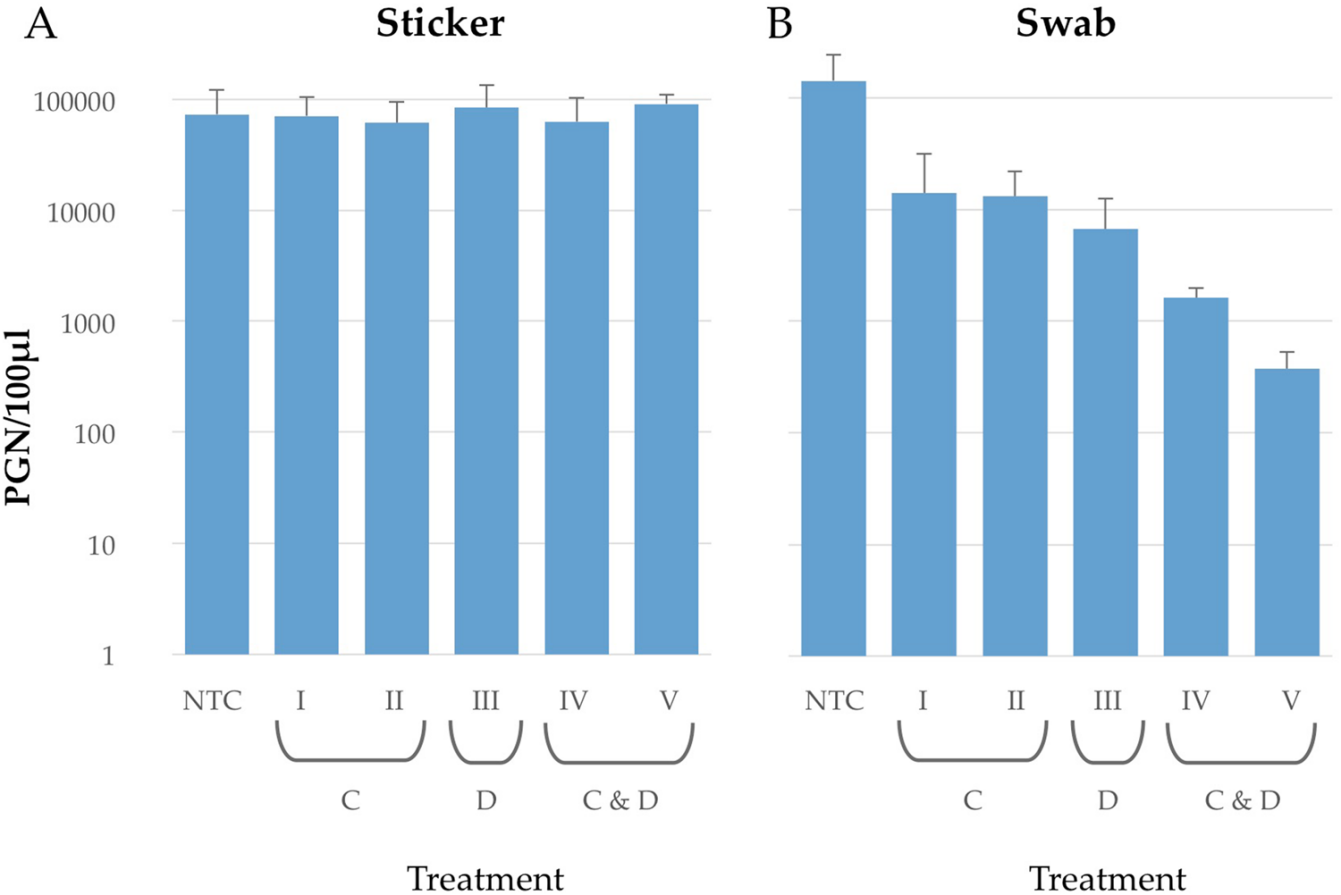
Viral genome recovery rate after cleaning and disinfection of paper-based stickers and cotton swabs. Surfaces or stickers applied to surfaces were artificially contaminated with a concentration of approximately 10^6^ PGN/100 µL. After drying, surfaces were treated with different methods, sampled, DNA extracted and analysed with qPCR. Treatments comprised a no treatment control (NTC), two cleaning treatments (C) (I) flush with water, (II) flushed with soapy water, the disinfection treatment (D) (III) wiped with Mirkozid and combined treatments (C and D) (IV) cleaning with BUDENAT ALKASEPT D 445 and wiped with Mikrozid, and (V) flushed with soapy water and wiped with Mikrozid. Each experiment was performed at least in triplicate and bars show mean values with respective standard deviations. Data were analysed using the Kruskal–Wallis test of independent samples.

As the paper-based stickers exhibited good linear and long-term monitoring properties, we quantified viral genomic material after implementation of various surface hygiene measures. Sterile ceramic tiles were used as experimental surfaces. Stickers were applied to the tiles and surfaces contaminated with known concentrations of virus solution. After a drying phase of at least 1 h, the surfaces were separately cleaned or disinfected with a variety of techniques. Five different hygiene measures were compared to cover as many routine procedures as possible, representing those commonly applied in food environments, hospitals and public places. Results presented in Fig. 3B reveal, not unexpectantly, that swabs were associated with viral information losses ranging across the different treatments. While both C: I and II cleaning measures led to a decrease of one log10 unit, the D: III disinfection measure resulted in a decrease of about 1.3 log10 units. The highest viral reduction was achieved with combined treatments C and D: IV and V, which were associated with reductions of 2–2.6 log10 units. In contrast to the swab results, sticker results in Fig. 3A show that the viral load remained constant over all investigated hygiene measures compared to the no treatment control (NTC). After direct comparison of each treatment as sticker versus swab, significant virus recovery differences between materials were observed after treatments (D: III), wiped with Mikrozid (*p ≤ 0.05), and C + D: IV, cleaning with BUDENAT ALKASEPT D 445 and wiped with Mikrozid (*p ≤ 0.05), and V flushed with soapy water and wiped with Mikrozid (***p ≤ 0.001). Treatment V was most effective, which achieved a significance-value of **p ≤ 0.001.

Considering the use of virus-inactivating treatments and electrostatic binding of virus particles to the paper stickers, recontamination events associated with the sticker itself can be excluded. However, confirmation will necessitate further investigations, using a range of viruses^25^. Overall, results indicate that the sticker approach was not associated with information losses that might be attributed to common hygiene measures. Further, results indicate that sticker sampling offers new opportunities for the identification of viral transmission hotspots and could provide further insight into viral transmission routes not available through “snapshot” swabbing approaches.

## Materials and methods

### Virus

Bacterial virus P100, purchased as PhageGuard Listex P100 solution (Batch 12G26, Lot: 308; Micreos, Wageningen, The Netherlands) was used in this study. For enumeration and propagation of phage P100 the related bacterial strain L. monocytogenes EGDe (ATCC BAA-679) was used. The bacteria were grown overnight at 37 °C in tryptone soya broth (TSB) with 0.6% (w/v) yeast extract (Oxoid Ltd., Hampshire, UK). After overnight incubation, the bacterial culture was ten-fold diluted in fresh medium and incubated at 37 °C for 3–4 h to obtain a maximum number of viable cells in the logarithmic growth phase (log phase). For propagation of virus stock, virus solutions were used for plaque assay, according to the protocol of Kropinski et al.^26^. Plates with confluent lyses were overlaid with 5 mL TSB or saline-magnesium (SM) buffer (5.8 g NaCl, 2.4 g Tris HCl, 1.0 g CaCl_2_, 0.1 g gelatin, add 1.0 mL H_2_O, pH 7.5) and shaken for about 3 h at room temperature or overnight at 4 °C. Afterwards the medium was separated and centrifuged at 8000 rpm for 2 min. Finally, the supernatant was filtered (0.02 μm), aliquoted and stored at − 20 °C.

### Quantification of infectious virus particles on stickers

Virus P100 was used at stock concentrations of approximately 10^10^–10^8^ PFU/100 µL. The investigated range of about 10^8^–10^4^ PFU/100 µL was based on a study of Herzog et al.^27^, which investigated virus P22 recovery efficiencies over a broad range of virus concentrations. Virus titers of artificially contaminated stickers, swabs and positive control were determined using the Small Drop Plaque Assay^28^. An overnight culture of L. monocytogenes EGDe was tenfold diluted in SM-buffer^26,28^ and subsequently a tenfold serial dilution from the aqueous-phase was prepared in bacteria-containing buffer. After 30 min of incubation at 37 °C, 20 μL of each dilution were dropped onto a tryptone soya agar (TSA)-plate and incubated overnight at the strain-respective temperature of 37 °C. The next day plaques were counted and compared with untreated, positive controls. All plaque assay experiments were repeated at least twice in duplicate.

### Viral DNA extraction

Each sticker was detached with sterile tweezers and transferred into a 1.5-mL Eppendorf tube and overlaid with 200 µL sterile water and thoroughly vortexed. The same procedure was performed with each swab sample and all positive and negative controls. Subsequently the DNA of each sample was isolated using the commercially available NucleoSpin tissue kit (Macherey Nagel, Düren, Germany) in accordance with the user manual (6.5 Support protocol for genomic DNA and viral DNA from blood samples)^29^. All experiments included a positive control (untreated phage-solution) and were repeated at least twice in duplicate or individually on three different days.

### Numeration of viral genome using real time PCR (qPCR)

For determination of the viral load (PGN/100 µL), the isolated samples were analyzed with qPCR. The primers for virus P100 qPCR were chosen according to Fister et al.^30^ and qPCR assays were performed using the non-specific DNA-dye EvaGreen Dye (Jena Bioscience GmbH, Jena, Germany). One qPCR reaction of 25 µL final volume contained 2.5 µL of 10 × reaction buffer (Biozym Scientific GmbH, Hessisch Oldendorf, Germany), 1.2 µM of probe EvaGreen Dye, 200 µM of each dNTPs (dATP, dTTP, dGTP and dCTP) and 1 U of Taq DNA polymerase (Biozym Scientific GmbH, Hessisch Oldendorf, Germany). Each P100 primer (forward: AGCAGAGTTTGAAAAAATTGATGACTAC and reverse: TCGTCATGCGTGTTTCTATGC), purchased from Eurofins Genomics (Ebersberg, Germany), was inserted to reach a final concentration of 500 nM. All qPCR assays were performed in an Mx3000 real-time PCR thermocycler (Stratagene, San Diego, CA, USA) with cycling conditions of 2 min at 94 °C, 45 × (30 s at 94 °C, 30 s at 59 °C, 30 s at 72 °C)^30^. The average efficiency of this qPCR was 99.6% and the average Rsq was 0.997. All qPCR experiments were conducted as a PCR-duplicate and included a negative amplification control as well as a standard series for calculation of a standard curve, whereas the standard curve was used for quantification of the viral nucleic acids.

### Artificial contamination of stickers, swabs and controls

Commercially available text-marking stickers (Markierungspunkte Ø 8 mm, permanent, no. 3013 yellow and 3175 white; Avery of CCL Industries, Inc., Toronto, Canada) were applied to an aseptic Petri dish 100 mm × 15 mm (Corning Inc., New York, USA) using sterile tweezers. The transferred stickers were sterilized with UV-C radiation for at least 15 min, as previously described by Bobal and Witte et al.^18^. Subsequently stickers were artificially contaminated or attached to the surface of interest for further experiments. Stickers and swabs were then contaminated with 5 µL virus solution and dried for at least 1 h. For the positive control the equi-volume of virus solution was transferred directly to 200 µL water and DNA isolation was performed as described above (2.3. Viral DNA Extraction).

### Cleaning and disinfection of surfaces

Different test conditions were chosen for the surface experiments. Each sterile ceramic tile (5 cm × 5 cm) was artificially contaminated with 5 µL virus solution, whereas the sticker was applied onto the tile before contamination. After a drying phase of at least 1 h, the surfaces were treated with different cleaning and disinfection treatments: (NTC) without washing, (I) flush with water, (II) flush with soapy water, (III) wiped with Mikrozid, (IV) cleaned with BUDENAT ALKASEPT D 445 and wiped with Mikrozid, and (V) flushed with soapy water and wiped with Mikrozid. Unwashed tiles were used as positive, untreated controls. For the flush treatments, (I) with water and (II) soapy water, the tiles were flushed twice with 1 mL liquid, whereas the soapy water tile was additionally rinsed with 0.5 mL distilled water. The soapy water was prepared by diluting the household cleaner Exact AC (E. Mayr, Vösendorf, Austria) in water to provide concentrations commonly used for surface cleaning (0.001–0.002% as described in the product data sheet). For treatment (III), disinfection of surfaces, Mikrozid AF liquid (Schülke & Mayr, Norderstedt, Germany) was applied by wiping. Treatment IV), cleaning with BUDENAT ALKASEPT D 445 and wiped with Mikrozid, was provided as BUDENAT ALKASEPT D 445 (Buzil-Werk Wagner GmbH & Co. KG, Memmingen, Germany), which is recommended as a high alkaline disinfectant cleaning agent for surface cleaning in food environments. For this treatment 1 mL of a 5% BUDENAT ALKASEPT D 445 solution was added to the ceramic tile and incubated for 30 min. Subsequently the tiles were rinsed with 0.5 mL water and wiped with Mikrozid. The last treatment, treatment (V), comprised a combined treatment with treatments (II) and (III). After surface treatments the previously applied sticker was further treated as described in 2.3 Viral DNA Extraction. The swab sample was taken by moistening the cotton swab with 100 µL SM-Buffer and afterwards the moistened swab was rubbed over the whole surface for 10 s. Afterwards the samples were treated as described in 2.3 Viral DNA Extraction.

### Controls, calculation of recoveries and statistical analysis

Recoveries from all experiments, determination of the PFU/100 µL (plaque assay) and the PGN/100 µL (qPCR) results were compared with those from the relevant positive controls. Further, sticker results were compared with corresponding swab results. Each experiment was repeated at least twice in duplicate, enabling calculation of average recovery and standard errors to account for variation of the data across means. Data were analysed using the Kruskal–Wallis test of independent samples from stickers and swabs (*p ≤ 0.05, **p ≤ 0.01, ***p ≤ 0.001).

## Conclusions

Current knowledge of virus transmission routes and virus persistence is limited by imperfect viral particle detection and long-term monitoring strategies. Consequently, this study has evaluated a new and easy-to-use sampling material, the paper-based sticker. This sticker has already been investigated in respect of bacterial sampling^18^, but its application to virus monitoring has remained uninvestigated. Single-sided adhesiveness means that stickers are attachable almost anywhere, including previously “difficult to access” areas and collect viral particles that would have otherwise been transferred onto the surface. Paper-based stickers also exhibit characteristics particularly suitable for long-term surface monitoring of viruses, unlike to swabs, which are made for the determination of actual viral load on a surface. These include a good linear binding capacity in terms of viral particles and viral DNA for up to 14 days, and resilience to routine cleaning and disinfection. Stable recovery rates of viruses from surfaces have also been demonstrated after various cleaning and disinfection measures without information loss. It is also unlikely that the stickers will have fomite properties as bound viruses remained non-infectious. In the future, various improvement options are to be tested in order to guarantee stable virus recoveries. While initial studies raise optimism for a new approach to surface virus monitoring, further studies are required to ascertain its suitability across a range of viruses, including enveloped and RNA viruses.

### Patents

This article is intended to discuss, but not modify, Patent Application Publication No. WO 2020/182924 A1, titled ‘A Novel Sampling Method for Long-Term Monitoring of Microbes’, for the purpose of increasing the accessibility of the scientific content contained therein. Inventors: Martin Bobal, Anna Witte, Patrick Mester and Peter Rossmanith. Current Applicant: Merck Patent GmbH (Frankfurter Strasse 250, 64293 Darmstadt). Filed: 14 March 2019. Published: 17 September 2020. This patent application is in no way limited by the publication of this article. Readers interested in the specific intellectual property covered by this patent should review the patent at the link provided below. Readers may also contact the authors or Merck Patent GmbH for licensing information. https://patentscope.wipo.int/search/en/detail.jsf?docId=WO2020182924.

## Supplementary Information

Supplementary Information 1.
