# Supplementary material for: A new long-term sampling approach to viruses on surfaces
Source: Sci Rep. 2021 Sep 2;11:17545. doi: 10.1038/s41598-021-96873-9 (PMC8413268; doi:10.1038/s41598-021-96873-9)
Supplement: Supplementary file 1 — Supplementary Information 1. [file 41598_2021_96873_MOESM1_ESM.docx]

**A New Long-Term Sampling Approach to Viruses on Surfaces - Supplementary Data**

**Julia Sommer 1, *, Martin Bobal 1, 2, Birgit Bromberger 1, Patrick-Julian Mester 1 and Peter Rossmanith 1, 3**

1 Unit of Food Microbiology, Institute of Food Safety, Food Technology and Veterinary Public Health Department for Farm Animals and Public Health in Veterinary Medicine, University of Veterinary Medicine, Veterinärplatz 1, 1210 Vienna, Austria

2 Current address: Vetfarm and Clinical Unit of Herd Health Management for Ruminants, Department for Farm Animals and Public Health in Veterinary Medicine, University of Veterinary Medicine, Kremesberg 14, 2563 Pottenstein, Austria

3 Joint BioEnergy Institute, Lawrence Berkeley National Laboratory, Berkeley, USA

* Correspondence: Julia Sommer (Julia.sommer@vetmeduni.ac.at; Tel.: +43-25077-3529; Fax: +43-25077-3590)

| **Table S1.** Number of positive findings of eight sticker values and swab values has de-termined by plaque assay from four independent experiments performed in duplicate. Virus titer concentrations ranging from 10^8^ to 10^4^ were used for artificial, direct contami-nation of paper-based stickers and routine cotton swabs. |
| --- |
| ***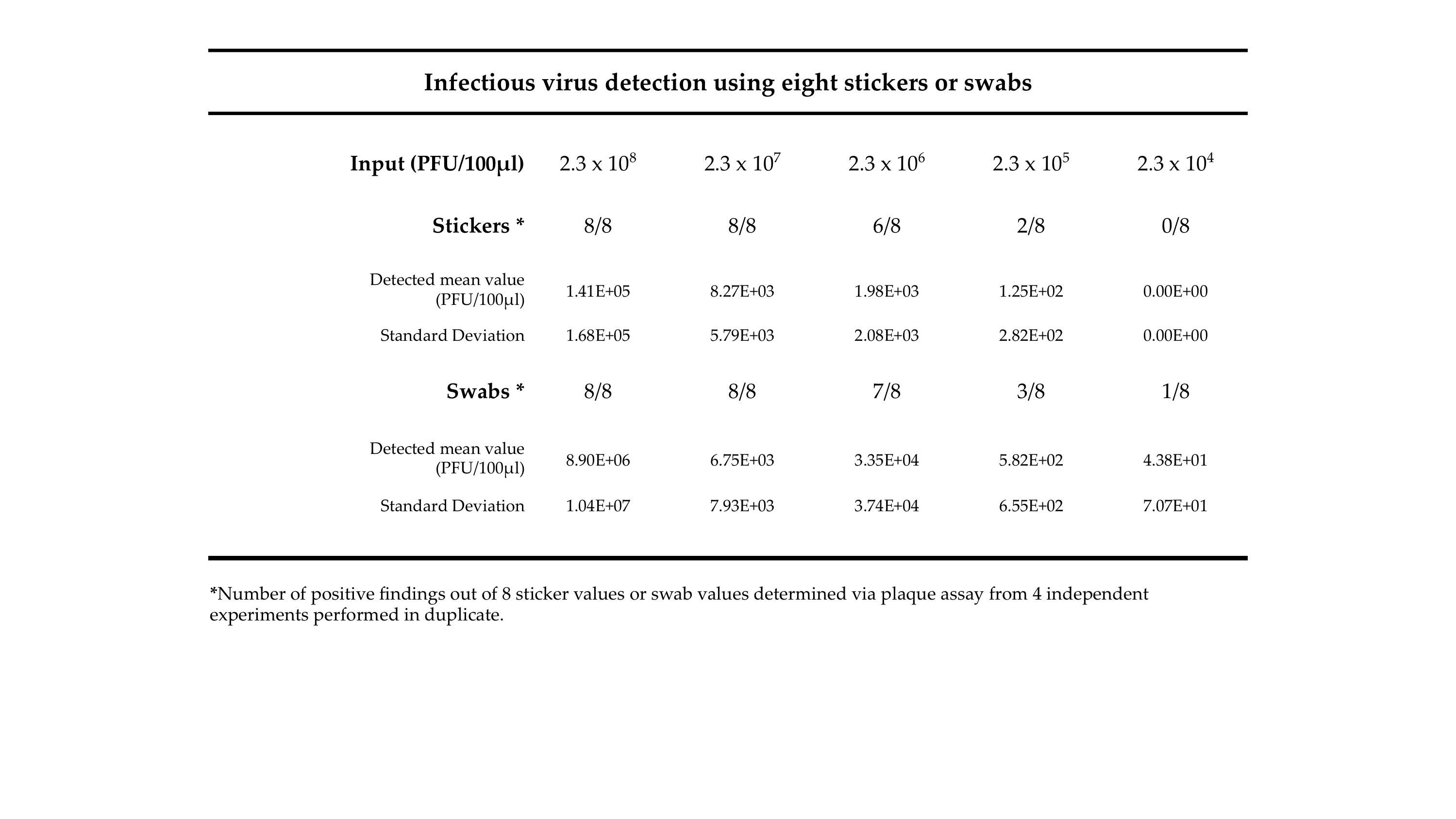*** |

| **Table S2.** Single values of linear recovery rates obtained from paper-based stickers and cotton swabs. |
| --- |
| 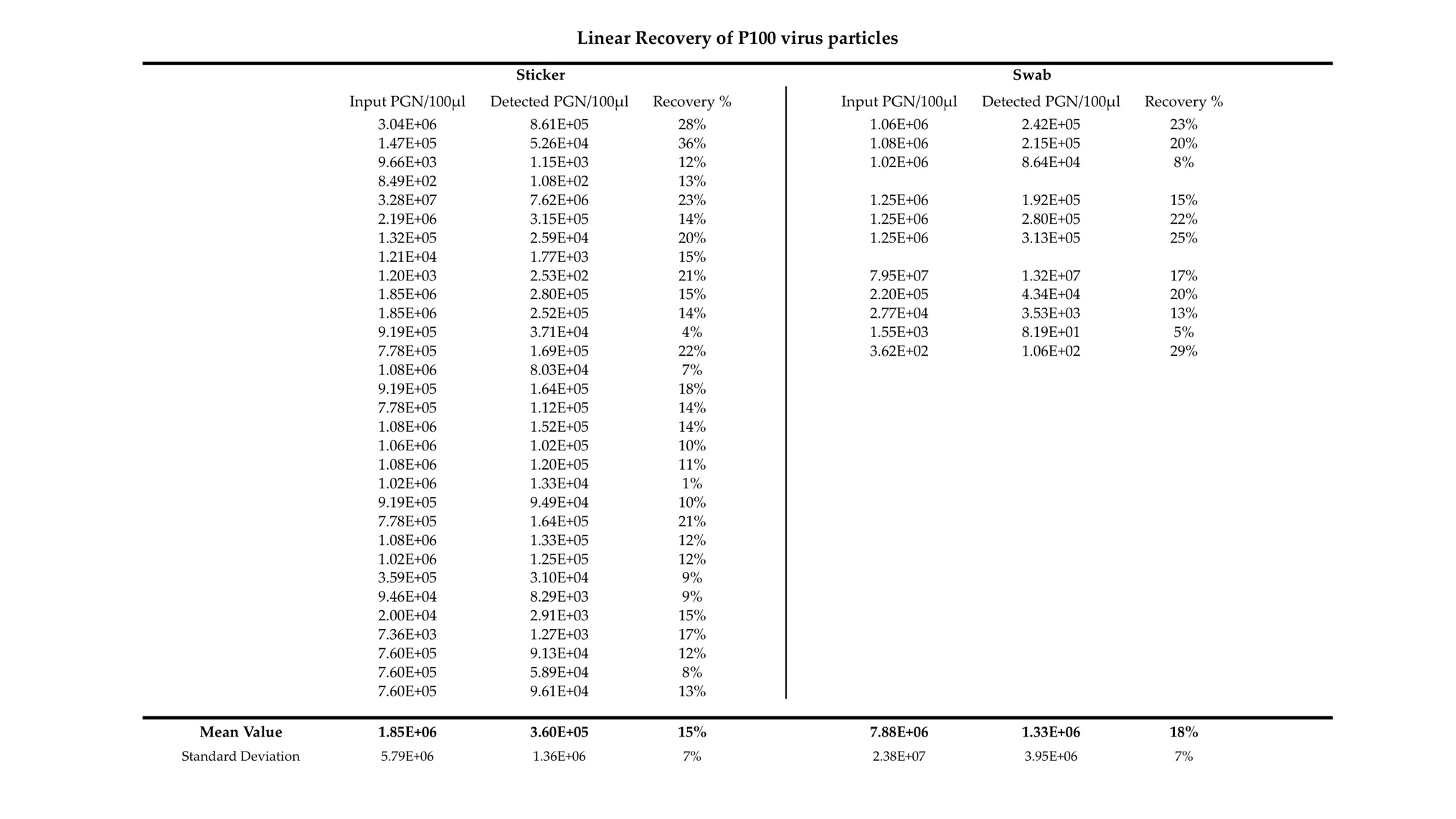 |

| 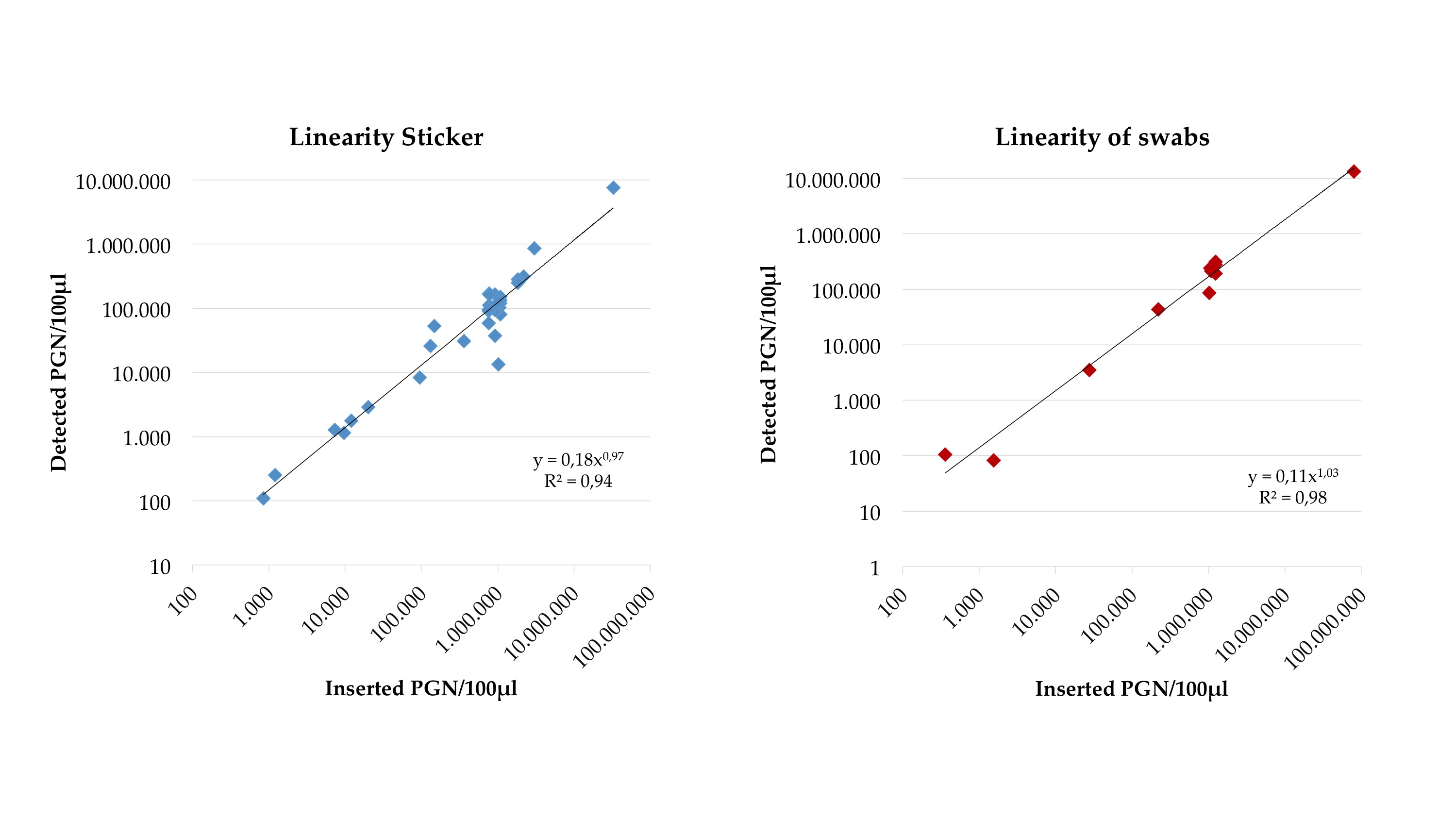 |
| --- |
| **Figure S1.** Quantification of artificially contaminated cotton swabs (n=11) with virus P100. Sampling materials were contaminated with virus titers ranging from 10^8^ to 10^2^ PGN/100µl to cover a broad recovery range. The x-axis plots the inserted amount of viral genomic material (PGN/100µl), while the y-axis plots recovered viral genomic material from samples. |

| 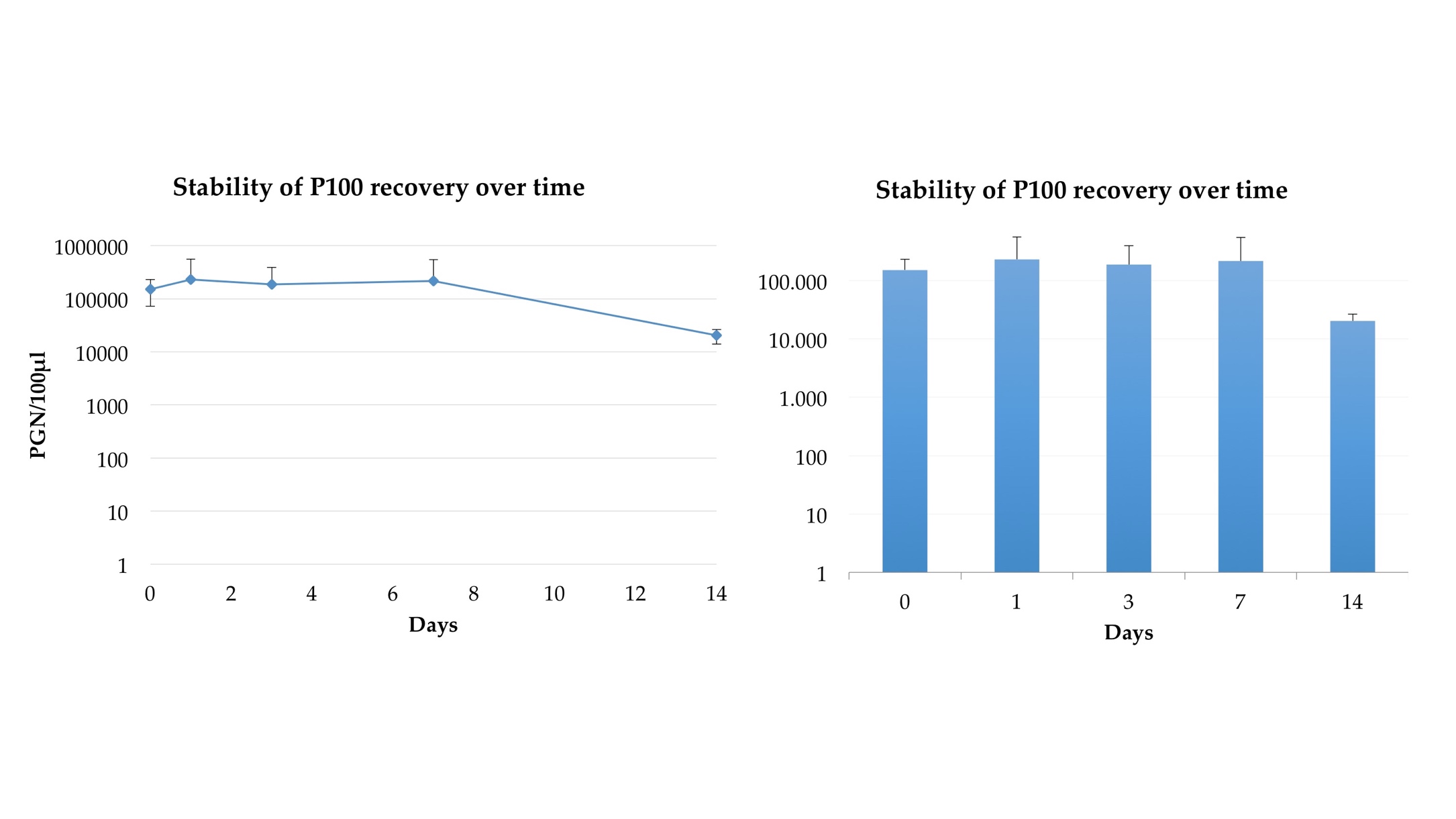 |
| --- |
| **Figure S2.** Overall stability of viral DNA on paper-based stickers over 14 days. Stickers were artificially contaminated with virus P100 and the viral load was determined after 0, 1, 3, 7 and 14 days of incubation at room temperature. Each experiment was performed, at a minimum, on two different days in triplicate. Bars illustrate mean values and respective standard deviations. |
